# Non-Aqueous Poly(dimethylsiloxane) Organogel Sponges for Controlled Solvent Release: Synthesis, Characterization, and Application in the Cleaning of Artworks

**DOI:** 10.3390/gels9120985

**Published:** 2023-12-15

**Authors:** Francesca Porpora, Luigi Dei, Teresa T. Duncan, Fedora Olivadese, Shae London, Barbara H. Berrie, Richard G. Weiss, Emiliano Carretti

**Affiliations:** 1 Department of Chemistry “Ugo Schiff” & CSGI Consortium, University of Florence, Via della Lastruccia, 3-13, 50019 Sesto Fiorentino, Italy; francesca.porpora@unifi.it (F.P.); luigi.dei@unifi.it (L.D.); fedora.olivadese@stud.unifi.it (F.O.); 2 Scientific Analysis of Fine Art, LLC, Berwyn, PA 19312, USA; 3 Department of Chemistry and Institute for Soft Matter Synthesis and Metrology, Georgetown University, 37th and O Streets NW, Washington, DC 20057, USA; ssl63@georgetown.edu (S.L.); weissr@georgetown.edu (R.G.W.); 4 Department of Scientific Research, National Gallery of Art, 2000 South Club Drive, Landover, MD 20785, USA; b-berrie@nga.gov; 5 National Research Council—National Institute of Optics (CNR-INO), Largo E. Fermi 6, 50125 Florence, Italy

**Keywords:** Polydimethylsiloxane (PDMS) organogel sponges, template effects, viscoelastic properties, solvent swelling/de-swelling, cleaning works of art

## Abstract

Polydimethylsiloxane (PDMS) organogel sponges were prepared and studied in order to understand the role of pore size in an elastomeric network on the ability to uptake and release organic solvents. PDMS organogel sponges have been produced according to sugar leaching techniques by adding two sugar templates of different forms and grain sizes (a sugar cube template and a powdered sugar template), in order to obtain materials differing in porosity, pore size distribution, and solvent absorption and liquid retention capability. These materials were compared to PDMS organogel slabs that do not contain pores. The sponges were characterized by Fourier-transform infrared spectroscopy with attenuated total reflectance (FTIR-ATR) and compared with PDMS slabs that do not contain pores. Scanning electron microscopy (SEM) provided information about their morphology. X-ray micro-tomography (XMT) allowed us to ascertain how the form of the sugar templating agent influences the porosity of the systems: when templated with sugar cubes, the porosity was 77% and the mean size of the pores was ca. 300 μm; when templated with powdered sugar, the porosity decreased to ca. 10% and the mean pore size was reduced to ca. 75 μm. These materials, porous organic polymers (POPs), can absorb many solvents in different proportions as a function of their polarity. Absorption capacity, as measured by swelling with eight solvents covering a wide range of polarities, was investigated. Rheology data established that solvent absorption did not have an appreciable impact on the gel-like properties of the sponges, suggesting their potential for applications in cultural heritage conservation. Application tests were conducted on the surfaces of two different lab mock-ups that simulate real painted works of art. They demonstrated further that PDMS sponges are a potential innovative support for controlled and selective cleaning of works of art surfaces.

## 1. Introduction

Recently, significant research has focused on the exploitation of the properties of some polymeric materials with porous structures (i.e., porous organic polymers, POPs) because they possess large surface areas and well-defined and tuneable pore size distributions [1,2]. The combination of these variable properties can provide synergic effects that are suitable for many technological applications [3]. POPs can be synthesised according to several routes with many functionalisations and are easily processed. Thus, the significant attention that POPs have received during the last decades can be attributed to their fundamental scientific characteristics as well as the extremely broad range of their realized and potential applications (these include greenhouse gas adsorption, proton conduction, energy storage and conversion, sensing, separation, catalysis, drug delivery and release, and tissue engineering) [3,4,5,6,7,8,9,10]. Polydimethylsiloxane (PDMS) organogel sponges [11,12,13,14,15,16,17,18,19,20,21,22,23,24,25,26,27] are important examples of POPs due to their elasticity, hydrophobicity, low surface tension, controllable porosity, low-toxicity, low-flammability, high thermal and electrical resistance, low bulk density, mechanical robustness, and high transmittance and low absorption of UV radiation [28,29,30,31,32,33].

A preliminary goal of this paper has been the assessment of a suitable strategy for the synthesis of PDMS-based systems using sugar as a templating agent [3,22,34] and exploiting the possibility of producing a large number of PDMS porous systems (i.e., sponges) [16,34]. Two different synthetic routes based on this strategy were followed. In one, sugar cubes with an edge of ca. 1 cm and comprised of grains with dimensions of a few hundred microns were chosen as templating agents; in another the templating agent was made from powdered sugar with grains having dimensions in the order of tens of microns. In both cases, the chemical and physicochemical properties of PDMS slabs (i.e., pure PDMS cast without templating agents) and of the two sponges (obtained using sugar cubes and fine powdered sugar as the templating agents) were investigated. Chemical analyses were performed using Fourier-transform infrared spectroscopy in the attenuated total reflectance mode (FTIR-ATR). Physicochemical characterizations were carried out using scanning electron microscopy (SEM) to obtain information about the morphology, and X-ray micro-tomography (XMT) was employed for the determination of the 3D structures and calculations of the porosity and of the pore size distributions. Analyses of these data have been useful for designing these materials for specific applications. Another aim of this work has been to obtain PDMS based systems with different porosity and pore size distributions that would present different and controllable properties in terms of solvent swelling, retention, and release. A possible application for such materials is as physically “gentle” cleaning agents for painted surfaces of historical and artistic interest. 

By exploiting (1) the ability to modulate the porosity of the PDMS systems by varying the granularity of the templating agent [3], and (2) the well-known compatibility of PDMS with a wide range of mid-to-low-polarity solvents [35], materials were produced with varying interactions between the systems explored and the organic solvents chosen here (many of which have been used for the cleaning of painted surfaces). These interactions were examined by measuring the amounts of the solvents absorbed [36] as a function of both solvent polarity and of the porosity of the PDMS). The rheology of the PDMS systems was investigated before and after loading solvents to obtain information about their effect on the viscoelastic properties, with particular attention to the elasticity and the complex viscosity of the systems. In fact, the ability to control these mechanical features is critical in determining two fundamental properties that a cleaning system tailored for painted surfaces of works of art should possess: (1) the retention of the solvents within the slabs/sponges and (2) adequate contact with the surface of the work-of-art. 

Despite the large differences among distinct classes of physical and chemical gels that have been tailored for the selective removal of coating materials on surfaces of painted works of art [37,38,39,40,41,42], few organogels and sponge-like systems have been reported for cleaning of works of art. Among these, a new class of polyhydroxybutyrate-based physical organogels have been prepared and successfully employed for the selective cleaning of bronze and water-sensitive painted surfaces of historical and artistic interest [43,44,45,46]. These systems can be easily prepared and loaded with organic solvents at different polarities (i.e., lactones and alkyl carbonates). The introduction of solvent-swelled PDMS gels adds to the range of solvents that can be applied via gel to works of art; moreover, the ability to tune the porosity implements a strategy that could potentially be applied to a range of gelled systems currently used in conservation. Thus, an aim of this research is a preliminary evaluation of the performance of PDMS-based water-free organogels. To investigate the use of these gels for conservation applications, cleaning tests were carried out on two lab mock-ups: a fresco and an easel painting that have naturally-aged, previously-applied surface coatings. 

## 2. Results and Discussion

**Figure 1:**
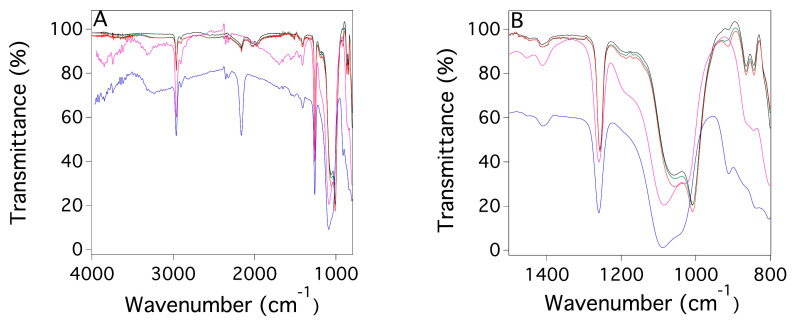
FTIR-ATR spectra of the A (magenta) and B (blue) components of the PDMS slab (ratio component A/component B = 20:1), the cross-linked polymer (black), and of the two different PDMS sponges (component ratio A/B = 10:1 for both systems); that with sugar cube as template (PDMS_SC red) and that with powdered sugar as template (PDMS_PS green). (**A**) reports the FTIR-ATR spectra in the range 4000–800 cm^−1^; (**B**) reports the FTIR-ATR spectra in the range 1500–800 cm^−1^.

The chemical characterization of the PDMS slabs (PDMS systems obtained without the use of any templating agent, see Section 4.2) and PDMS_SC (PDMS systems obtained using a sugar cube as templating agent, see Section 4.2) and PDMS_PS sponges (PDMS systems obtained using powdered sugar as templating agent, see Section 4.2) using FTIR-ATR spectra are reported in Figure 1. All of the characteristic peaks of both the two components, the ‘base’ A and ‘curing agent’ B, and the cross-linked polymer are in agreement with the data reported in the literature [47,48]. Moreover, no meaningful differences in the profiles of the FTIR spectra of the three PDMS-based systems are observed, indicating that their chemical compositions are the same and that the templating agents have no influence on that. The absence of signals associated with sugar suggests that no detectable residues of the sugar templating agent are present. In addition, no glucose was detected by a Fehling [49] test carried out on the central portions of PDMS_SC and PDMS_PS fragments.

**Figure 2:**
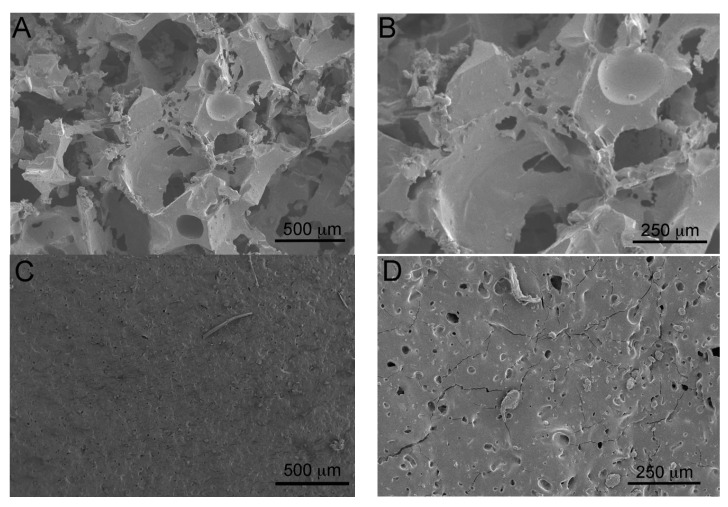
SEM micrographs of two different PDMS sponges: (**A**) PDMS_SC magnification 50×; (**B**) PDMS_SC magnification 100×; (**C**) PDMS_PS magnification 50×; (**D**) PDMS_PS magnification 100×. For both samples, the ratio of components A/B was 10:1.

The morphology of the two porous sponges was investigated by SEM. Figure 2 shows the micrographs of the two PDMS sponges, PDMS_SC (A and B) and PDMS_PS (C and D).

The images in Figure 2 indicate that the two templating agents result in very different morphologies, with a porosity much higher for sponges synthesized using sugar cubes than for systems obtained using the powdered sugar template.

**Figure 3:**
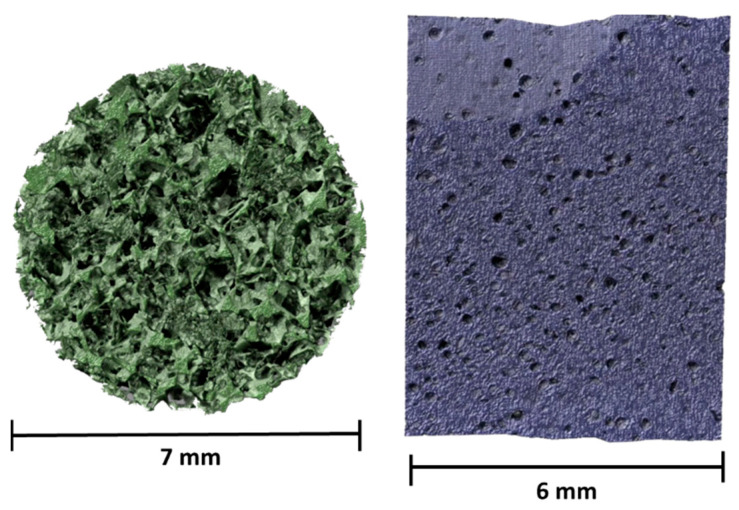
XMT 3D images for two different PDMS sponges in which the A/B component ratio was 10:1: (**left**) PDMS_SC; (**right**) PDMS_PS.

**Figure 4:**
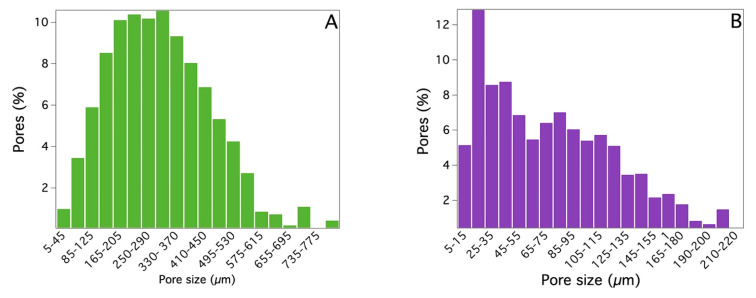
Histograms of the pore size distributions for two different PDMS sponges (10 × 5 × 5 mm) derived from XMT measurements: (**A**) PDMS_SC; (**B**) PDMS_PS. For both samples, the A/B component ratio was 10:1.

To obtain more detailed information about the porosity of the two systems, XMT measurements were carried out, and the 3D images obtained through the procedure described in Section 4.2 are displayed in Figure 3. These images confirm that the use of the two different templating agents produces sponges with different porosities [16,34]. By employing the procedure described in Section 4.3, it was possible to deduce the pore size distribution (for pores larger than 5 μm) for both sponges. These values are reported in Figure 4A,B for PDMS_SC and PDMS_PS, respectively. The results confirm that the pore size of the PDMS_SC (ca. 303 μm) is larger than that of PDMS_PS (ca. 76 μm). Table 1 summarizes the results from the porosity analysis performed on the two sponges.

**Figure 5:**
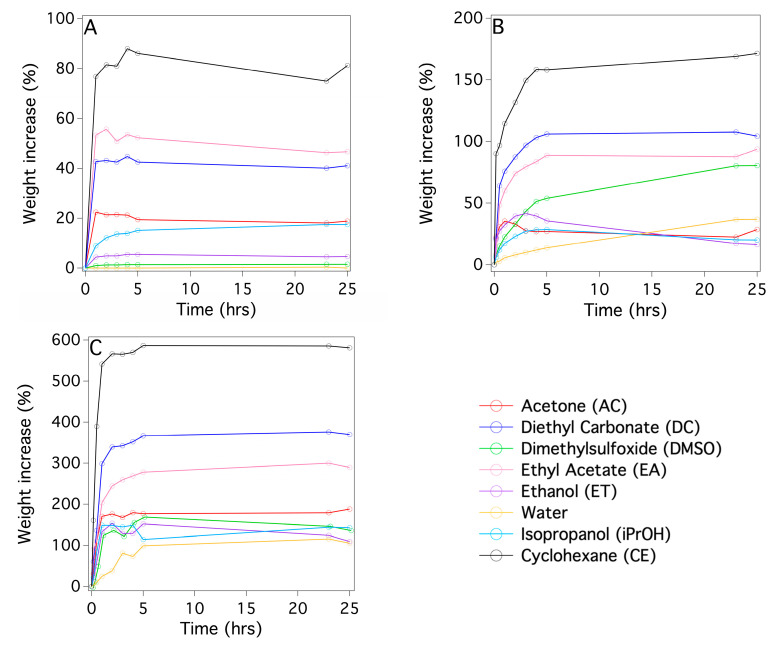
Kinetics curves for solvent absorption of the two different PDMS sponges and of the PDMS slab for various solvents (ratio component A/component B = 10:1): (**A**) PDMS slab, (**B**) PDMS_PS, (**C**) PDMS_SC.

A fundamental aspect to investigate here, in view of a possible application of these systems as cleaning tools for painted surfaces, is a comparison of solvent absorption by the two sponges and that of the PDMS slab, focusing on both the swelling of the slab and on the capillary action of the porous PDMS sponges. This experiment is fundamental to understand the maximum amount of solvent that can be loaded into the three different systems in the view of a possible application as cleaning tools for painted surfaces; this is the reason why the absorption tests have been carried out over up to 25 h (Figure 5).

**Figure 6:**
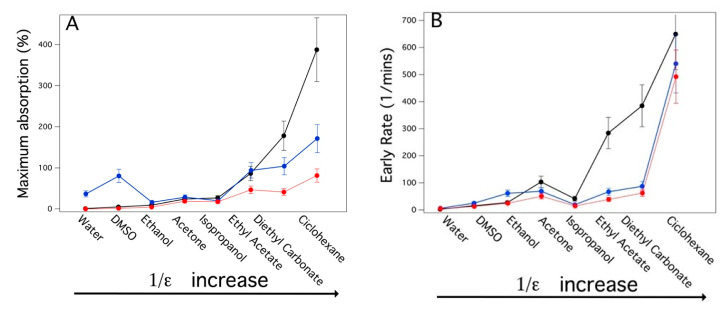
Maximum amount of organic solvent absorbed (**A**) and swelling rate (**B**) by PDMS slab (red dots), PDMS_PS, (blue dots), PDMS_SC (black dots). The solvents on X axis are presented as a function of 1/ε, where ε is the dielectric constant of the solvents.

In that regard, Figure 5 shows the kinetics of absorption by the three systems with various organic solvents, including some that are used for cleaning purposes in cultural heritage conservation. Figure 6A,B report the maximum amounts of the solvents absorbed by the PDMS-based systems and the initial absorption rates, respectively. From Figure 5 and Figure 6A, the PDMS_SC, with the highest porosity and the larger pore size, absorbed up to four times the amount of solvent as absorbed by the PDMS_PS sponge with a higher rate (Figure 6B). This indicates that porosity is the key factor in determining the kinetics of solvent absorption. This is also confirmed by the lowest absorption amount is observed for the nonporous PDMS slabs (Figure 5A). Another result, that is in agreement with the literature data [35], is the strong dependence between the rates of the absorption and the magnitude of the dielectric constants ε of the solvents (Figure 6A,B). As expected, because PDMS is a low-polarity polymer, the maximum absorption amount and rate were observed for solvents with low ε values, such as cyclohexane; conversely, in polar solvents such as water, ethanol and DMSO, absorption was smaller and slower. This behavior is due to the large difference between the physico-chemical affinities of the solvents and the hydrophobic network of the PDMS matrix. Absorption tests have been carried out with cyclohexane and DMSO, solvents that should not be used in conservation due to health concerns, in these experiments to demonstrate that it is possible to load solvents having very different polarities into the PDMS based systems.

**Figure 7:**
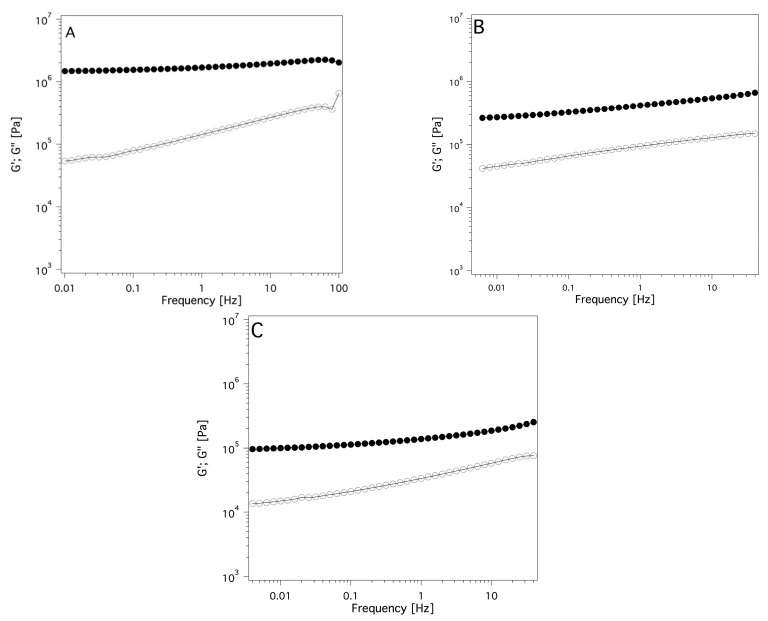
Frequency sweep diagrams of the (**A**) PDMS slab, (**B**) PDMS_PS, (**C**) PDMS_SC systems. Filled circles indicate G′; open circles indicate G″ (component ratio A/B = 10:1).

**Figure 8:**
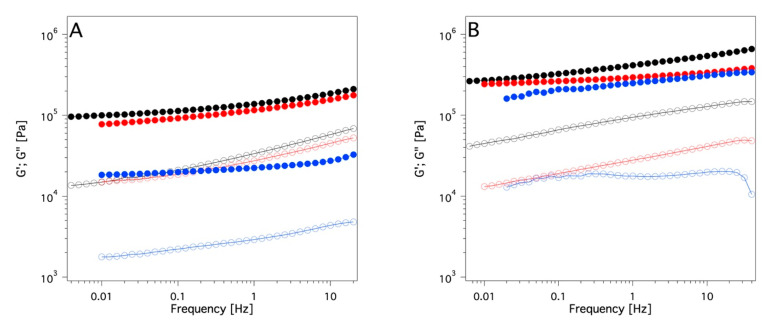
Frequency sweep diagram of the: (**A**) PDMS_SC sponge alone (black), with absorbed DC (blue), with absorbed DMSO (red); (**B**) PDMS_PS sponge alone (black), with absorbed DC (blue), and with absorbed DMSO (red). Absorption time: 24 h. Filled circles indicate G′; open circles indicate G″.

Rheological measurements were carried out to obtain information about the viscoelastic properties of the systems. These data indicate a solid-like rheological behavior for all the investigated samples (G′ >> G″ over the whole range of frequencies investigated; see Figure 7), and, according to the literature, [50] they can be mechanically classified as solid-like materials. Since one of the main goals of the study is the application of these systems as cleaning tools for works of art surfaces, it was crucial to ascertain how the viscoelastic properties could be affected by the presence of absorbed solvent in the swollen sponges. Therefore, frequency sweeps were carried out after the absorption of some organic solvents that were chosen among the ones used for cleaning purposes in cultural heritage conservation. Four solvents covering an extended range of polarities, were selected: ET, DMSO, DC, and EA (acetone was not used here due to its low boiling point). Figure 8, as an example, reports the trend of G′ and G″ versus frequency sweep for the two sponges containing two solvents, among the four tested, at the extremes of the polarity scale, that are DC and DMSO. The other frequency sweep diagrams of PDMA_SC and PDMS_PS loaded with ET and EA are reported in Figure S1. These data clearly indicate that none of the four solvents tested alters the porosity or the gel-like nature of the two sponges independently (see Figure 7 and Figure S1).

**Figure 9:**
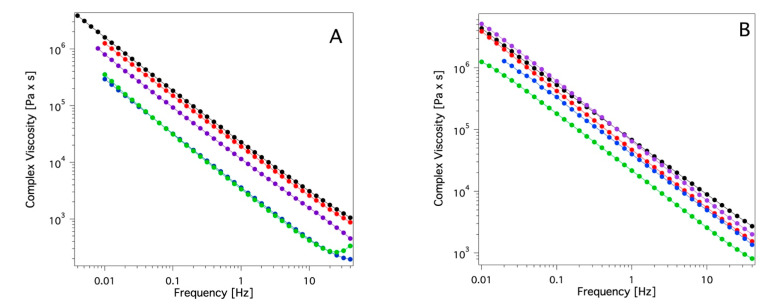
Complex viscosity of the PDMS_SC (**A**) and PDMS_PS (**B**) sponges alone (black) and with absorbed ethyl acetate (green), diethyl carbonate (blue), DMSO (red) and ethanol (purple).

The main finding from the rheology measurements (Figure 8 and Figure S1) is a decrease of the G′ value for both the investigated systems (PDMS_SC and PDMS_PS) after loading the four solvents. This effect is higher for DC than for DMSO and, with the same solvent this effect is more pronounced for the PDMS_SC. This is attributable to the higher porosity of the PDMS_SC system that allows the absorption of a larger amount of this solvent (Figure 5B,C). The trend of the complex viscosity η* (see Figure 9A,B) for the pure sponges and the swollen sponges by the four solvents after 24 h of absorption confirms the findings of frequency sweep measurements: a pronounced decrease of η* due to the presence of a purely viscous liquid phase within the pores is observed. Moreover, this effect seems to be inversely proportional to the polarity of the confined solvents as indicated by the lowest η* values of the DC and EA-based systems. 

**Figure 10:**
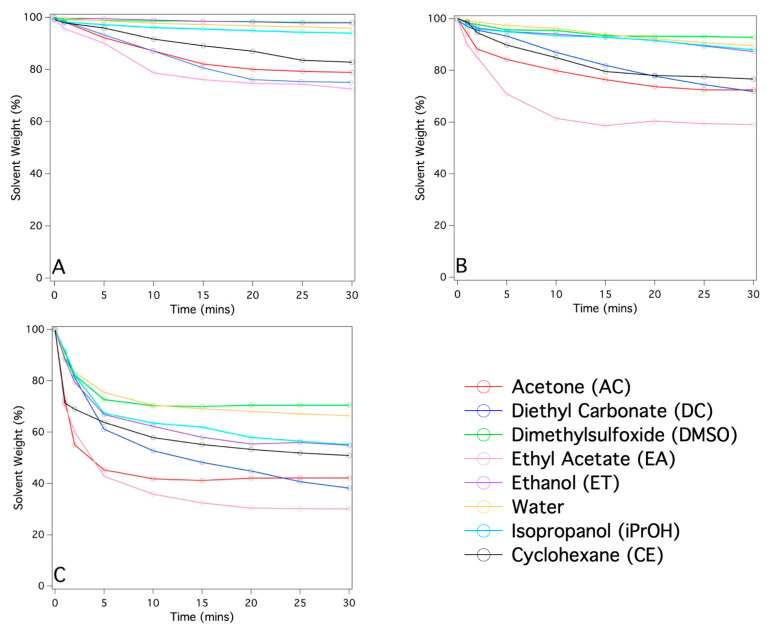
Kinetic curves describing the weight loss of the PDMS based systems soaked with organic solvents and placed in direct contact with a stack of Whatman^®^ papers: (**A**) PDMS slab, (**B**) PDMS_PS, (**C**) PDMS_SC. The A/B component ratio is 10:1.

From an application standpoint, one of the main advantages of using gels for cleaning surfaces of porous matrices of historical and artistic interest is linked to the high retention of the solvents confined within them. This allows control of the cleaning action which ideally remains limited to the contact area between the gel itself and the surface of the object to be cleaned. To obtain information for the PDMS-based systems investigated here, absorption tests of the solvents loaded inside them were carried out on sheets of Whatman^®^ paper (which was chosen as a model porous system). The results are reported in Figure 10.

The data reported in Figure 10 clearly indicate that the extent of solvent release is proportional to the porosity of the PDMS-based networks and, therefore, to the maximum amount of solvent that can be loaded inside them. In fact, for the PDMS-SC system, release values ranging approximately between 30 and 65 wt% are observed, while for the PDMS slab and PDMS-PS, the values never exceeded 50 wt%.

**Figure 11:**
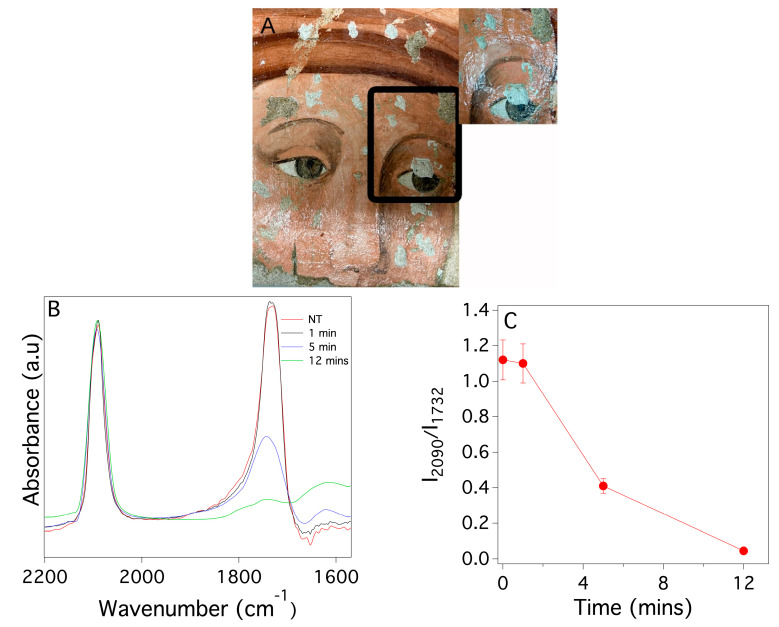
Results of the cleaning test carried out on a fresco mock-up coated with a surface layer of naturally aged poly(EMA/MA) 70:30 by means of a PDMS_PS sponge loaded with ca 10 wt% of EA: (**A**) image of the mock-up before (big picture) and after the cleaning (small picture showing a detail of the area where the 12 min test was carried out): (**B**) FTIR spectra of the resin extracted from mortar fragments collected in the area where the cleaning tests were carried out before (NT, red line) and after the PDMS_PS system application with different contact times: 1 min (black line), 5 min (blue line) and 12 min (green line). (**C**) ratio between the intensity of the peaks at 1732 cm^−1^ and at 2092 cm^−1^ as a function of the application time.

The efficacy of the PDMS based systems as cleaning agents for painted surfaces was first tested on the surface of a fresco mock-up with a 20-year-old surface layer of poly(EMA/MA) 70:30. Figure 11A shows a region of the painting affected by this surface coating. Different tests were carried out by increasing the contact times up to 12 min. The grazing-light image on the right of Figure 11A, collected after the application of a PDMS_PS loaded with EA (ca. 10 wt%) for 12 min, shows the complete disappearance of the glossy effect typical of the copolymer film in the area of the cleaning test. This image indicates macroscopically that the cleaning system was effective for removing the aged coating. Figure 11B reports the FTIR spectra of the organic fraction extracted from mortar samples collected from areas of the fresco where the cleaning tests was carried out (for the detailed experimental procedure, see Section 4). In particular, the FTIR analysis of the cleaned surface (see Figure 11B) confirmed that the poly(EMA/MA) 70:30 layer was no longer detectable after a 12 min treatment with the PDMS_PS sponge, based on the absence of the peak at 1735 cm^−1^, which is a marker of poly(EMA/MA) 70:30. Furthermore, Figure 11C shows the trend of the ratio between the intensity of the peaks at 1732 cm^−1^ and 2092 cm^−1^ (stretching of the C≡N bond of the Prussian blue used as internal standard) as a function of the application time. The decrease in the value of this parameter indicates the progressive cleaning action of the PDMS_PS sponge by increasing the contact time with the fresco surface.

The surface morphology was investigated by scanning electron microscopy (SEM); it is typical of wall paintings affected on the surface by polymeric coatings that make the surface “smooth” (i.e., with very low roughness and open porosity (Figure S2A)). After application of the cleaning system, the texture of the surface appeared much rougher with a higher porosity that is typical of an original mortar (Figure S2B).

**Figure 12:**
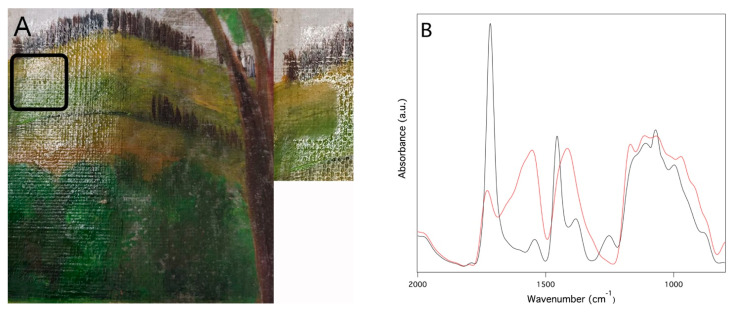
Cleaning of a canvas painting mock-up by means of a PDMS_PS system loaded with about 10 wt% of EA. (**A**) Image of the mock-up before and after the cleaning (small insert on the right) showing a detail of the area where the test was carried out; (**B**) ATR-FTIR collected in the cleaning test area before (black line) and after (red line) the application of the PDMS_PS system.

Another potential application for these systems is the removal of aged organic coatings from the surface of easel paintings, as shown in the case study involving the removal of a 25 year naturally aged polymeric ketone resin varnish from the surface of a canvas mock-up. Even in this case, the cleaning test was carried out by means of a PDMS_PS loaded with EA (ca. 10 wt%) with a contact time of 5 min. In fact, from the Teas diagrams [51] it can be deduced that EA is a good solvent for the removal of this class of varnishes. Figure 12A shows the area of the painting treated. The picture on the right, collected after the application of the PDMS_PS system, indicates the visual absence of the varnish layer in the area involved in the cleaning test. ATR-FTIR spectra of the artwork surface before and after cleaning are consistent with this conclusion: the cleaning (Figure 12B, black line) shows strong absorbances centered at 1716 cm^−1^, 1454 cm^−1^ and 1070 cm^−1^ attributable to the ketone resin [52]; in the spectrum collected after the cleaning test (Figure 12B, red line), the intensity of these diagnostic peaks is strongly decreased, indicating the removal of the surface coating. Also, SEM analysis (Figure S3) of the treated area confirms qualitatively that the cleaning test was successful. Note that the morphology of the paint surface after the cleaning test (Figure S3C) is similar to that of the area of the mock-up where the surface coating was absent (Figure S3A) and that it appears less homogeneous and rougher than the region treated by the surface varnish layer before the application of the PDMS_PS system (Figure S3B). 

In both cases FTIR spectra did not indicate the presence of PDMS residues on the cleaned surfaces.

The first aspect to be discussed is the two synthetic routes selected to obtain the two POPs PDMS that have different total porosity and pore size distributions. Although the results are consistent with results already reported in the literature for using a sugar cube [34], the material prepared using powdered sugar was more compact with much lower porosity. The FTIR-ATR analysis reported in Figure 1 indicates that there is no meaningful difference in the composition of the PDMS sponges with respect to non-porous slabs (i.e., that no sugar residues were present).

Thus, for these three systems, the non-porous PDMS slab, porous PDMS_SC and PDMS_PS, we conclude that the chemical nature of the materials is the same and the templating agents did not leave any discernible residues. 

From a comparison of the morphology and porosity data reported in Table 1 and Figure 3 and Figure 4, it is evident that the two sponges, although chemically the same, have different porosities: PDMS_SC has a much higher porosity than PDMS_PS derived from the large dimensions of the grains constituting the sugar cube (in the order of few hundreds of microns). Moreover, the mechanism of polymerization in the presence of the two templating agents (sugar cube or powdered sugar) differed. In the first case, the appropriately mixed A and B components of the polymerization were absorbed by the porous structure of the sugar cube, so that the polymerization occurred within the fixed macropores of the sugar cube. In the second case, the polymerization occurred at the random interstices among the fine sugar grains that were mixed with the A and B components. Both the different size of the crystal grains for cube and powdered sugar and the larger macropores present in the cube with respect to the smaller ones, consisting of interstices among fine powder grains, generated a large difference in the two PDMS sponges. 

The swelling kinetics of the PDMS slab, PDMS_SC and PDMS_PS sponges was a clear indicator of the large difference in porosity among the three materials. Figure 5 shows that PDMS_SC has both the fastest kinetics of solvent absorption and the largest amount of swelling at the asymptotes. This was ascribed to its very high porosity with respect to PDMS_PS and to the non-porous PDMS. In this regard, we reiterate that solvent absorption by the polymer was due simply to swelling phenomena for the PDMS slab but, two synergistic effects were present (i.e., swelling of the polymer enhanced by capillary action mediated by the pores) for the POPs PDMS sponges. These results agree with observations from the literature [35] which show that the driving factor for solvent absorption by PDMS is the magnitude of the hydrophobic interaction which is largest for solvents with the lowest dielectric constants (Figure 5). This is a key point in view of a possible use of these systems as cleaning tools for surfaces of works of art. The swelling/absorption data indicate that these materials may be suitable as cleaning solvents which are able to be released in a controlled way which minimizes both their spreading into the work of art porous support, and any water contamination that is typical for most of the commonly used chemical hydrogels.

Another aspect investigated is their rheological properties (reported in Section 4 and in Supplementary File data). All the investigated systems are characterized by a solid-like behavior, so it is possible to classify them as chemical organogel sponges. As far as we know, these systems may be truly innovative tools for cleaning works of art, especially where even the presence of traces of water during the cleaning procedure represents a possibility of serious damage for the surface. A further interesting finding was that the amount of solvent absorbed by the PDMS-based systems did not meaningfully change the viscoelastic behavior of the PDMS sponges and slabs. As a result, the application tests could open new perspectives for cultural heritage conservation in the field of selective cleaning. 

In that regard, the action of the PDMS_PS system loaded with the 10 wt% of acetone, and two different polymeric coatings present on the surface of two lab mock-ups—poly(EMA/MA) 70:30 and a ketone resin on the surface of a fresco and of a canvas painting respectively—indicates the selective extraction of these molecules within the cleaning agent. FTIR analysis carried out on the painting surface before and after the application of the cleaning systems clearly highlights the removal of both the coatings and the recovery of the original morphology typical of uncoated paint layers. Moreover, once the cleaning action is carried out, the PDMS_PS systems have been completely removed, without leaving any instrumentally detectable traces on the cleaned area. This avoids both the use of free-flowing organic solvents and any mechanical action on the paint surface. This feature represents a fundamental improvement over many other the traditional cleaning methods that employ physical gels [53]. 

## 3. Conclusions

Synthetic routes have been employed to obtain two types of PDMS sponges with large differences in both their total porosity and mean pore size. These sponges show strong promise as systems to be tried in cleaning tests for the surfaces of works of art. They allow the roles of porosity and pore size in determining solvent/release retention, its spreading within the works of art support, and the solubilization ability against coatings to be removed.

Chemical characterization by FTIR-ATR permitted a comparison of the properties of PDMS slabs with the above-mentioned sponges and to ascertain that the chemical composition of the cross-linked polymer was the same for all the investigated systems. Moreover, no traces of the sugar templating agents used for the preparation of the sponges were present after polymerization. Physicochemical characterizations, carried out by means of SEM, XTM, and porosity determinations, showed that while the sponges obtained using sugar cubes as a templating agent have a porosity around 77% with a mean pore size ca. 300 μm (with a distribution of pore diameters ranging from a few microns to several hundred microns), the porosity of the sponges synthesized using powdered sugar decreased up to 10% with a mean pore size of ca. 75 μm. In this case the distribution of pore diameters ranges from a few microns up to 200 microns. 

The very low polarity of PDMS is a key factor in determining the amount of absorbed solvent through polymer swelling and capillary action. They decrease as the dielectric constant of the liquid is increased. 

The rheology data indicate that all the systems tested are characterized by a gel-like behavior. G′ was larger than G″ over the measured frequency range. In materials with higher porosity, the large amount of bulk and free solvent entrapped within the large pores caused a decrease of G′ compared to the dry sponge. This effect is strictly related to the polarity of the solvent according to the greater absorption of low polarity solvents within the pores due to the high hydrophobicity of the PDMS. At lower porosities, the mean sizes of the pores were strongly reduced, and the bulk and free solvent absorbed by the porous polymers were strongly decreased. As a result, the effect on the elastic behavior of the sponges was limited too. 

Application of the two porous PDMS based systems containing a small amount of a solvent commonly used for the removal of foreign patinas from painted surfaces of historical and artistic interest (i.e., acetone) allowed selective removal of aged varnishes from the surfaces of two mock-ups that simulate artworks with different substrates (i.e., a fresco and a canvas), without negatively altering the original substrates and without leaving any discernible traces of the PDMS_PS system. These preliminary results/proofs of concept, indicate that the PDMS systems loaded with different organic solvents presented in this paper should be explored further as selective cleaning tools for painted surfaces of historical and artistic interest, even for the cleaning of paintings having different compositions (i.e., modern and contemporary paintings).

The systems presented in this paper are complementary to other physical organogels presented in the literature [43,44,45,46]. In particular, due to their physicochemical properties, the PDMS-based organogels and sponges can be loaded with solvents (tunable by varying the contact time between the sponge and the solvent to be loaded, see Figure 5) having different dielectric constants (varying from 2 for cyclohexane to 47 for DMSO). Moreover, thanks to their strong cohesive internal forces (i.e., covalent bonds) they minimize residues left onto the painted surface as indicated by the FTIR data collected on the surfaces after cleaning.

Moreover, the PDMS-based organogels and sponges represent a potentially important alternative to the most used physical cleaning techniques, such as laser or micro-sandblasting, especially in those cases where the cleaning operation must have a high selectivity and a gentle mechanical action.

## 4. Materials and Methods

### 4.1. Materials

Polydimethylsiloxane, Sylgard 184^®^ from Dow Corning Corporation (Wiesbaden, Germany), was composed of the base (component A) and a curing agent (component B). Commercial sugar cubes (1 × 1 × 0.7 cm, Dietor Vantaggio^®^) were pure saccharose grains having average dimensions of about 200 μm and the powdered sugar (dimensions of the grains on the order of tens of microns) were supplied by Sperlari S.r.l., Cremona, Italy. Solvents were from Sigma-Aldrich c/o Merck Life Science S.r.l., Milano, Italy (purity > 99%) and used without further purification. Dow Sylgard™ 184 was supplied by DOW Italy, Milan, Italy. Water was purified by a Millipore MilliQ Direct-Q^®^ & Direct-Q UV water purification system (Water Resistivity: 18.2 MΩ at 25 °C) purchased from Merck-Millipore, Milano, Italy. Polyethyl methacrylate/methyl methacrylate 70/30 (ParaloidB72^®^) was purchased from Zecchi, Firenze, Italy. The ketone resin was purchased from Phase s.r.l., Firenze, Italy. Melinex® was purchased from Phase s.r.l., Firenze, Italy. Watman® paper was purchased from Cytiva, Little Chalfont, Kent, UK.

### 4.2. Synthesis Methods

The best ratio to obtain a sufficiently elastic material for applicative purposes was found to be 10:1 A/B by weight. The synthesis was performed as follows:

1. a total amount of 2 g of A and B were vigorously stirred in a Petri dish for approximately 2 min;
2. after homogenization, the Petri dish was placed in a dryer under vacuum at room temperature (ca. 20 °C) to eliminate air bubbles;
3. the polymerisation occurred at room temperature (ca. 20 °C) and soft filter paper was placed onto the Petri dish to avoid dust deposition;
4. after three days, the resulting material, transparent to the eye and elastic, was formed (see Figure S4).

The analogous procedure was performed inside an oven at 60–80 °C for 2 h instead of at room temperature for 3 days. The scheme of the reaction is reported in the literature [54,55,56].

The same procedure was used also for the synthesis of the porous sponges by using the sugar template method [34] with a further step (also for both the PDMS-based sponges, PDMS_SC and PDMS_PS, the A:B ratio was chosen as 10:1). For the sugar cube sponges (PDMS_SC), the synthesis procedure is reported below:

1. after air bubbles were eliminated, a sugar cube (1.4 g and 1 × 1.5 × 1 cm composed of grains having dimensions ranging in the order of 150–300 μm) was put onto the A/B mixture (A:B ratio was 10:1) at the center of the Petri dish;
2. the Petri dish containing the A/B mixture and the sugar cube was put into a dryer under vacuum for 30 min at room temperature (ca. 20 °C) and then kept under static vacuum for 2 h to allow complete infiltration of the A/B mixture within the porous structure of the sugar cube;
3. then, the Petri dish was put into an oven at 60 °C for 2 h. The curing time was determined by monitoring the trend of the value of the elastic modulus G′ at 60 °C as a function of the time for 3 h at constant frequency (1 Hz) and amplitude strain (1%). Figure S5 shows that even after 1.5 h G′ reached an asymptotic value indicating that the polymerization reaction was complete.
4. After this time, it was possible to put the sugar cube imbibed with PDMS into a vial filled in with MilliQ water at 40 °C to entirely solubilize the sugar and obtain only the porous PDMS_SC sponge (see Figure S6).

For sponges made using a powdered sugar as template (PDMS_PS), the synthesis procedure is reported below:

1. 2.8 g of powdered sugar (composed of grains having dimensions of a few tens of microns) was incorporated within the A/B mixture until it became a homogeneous system and was placed in a silicon mold (1 × 1.2 × 1.5 cm);
2. the mold was put inside a Petri dish and dried under vacuum for 30 min to eliminate any air bubbles;
3. thereafter, it was put into an oven at 60 °C for 2 h to complete the polymerization.
4. it was possible to remove the powdered sugar by keeping the sample (PDMS_PS cube) for 15 h at 90 °C in a beaker filled with MilliQ water to entirely solubilize the powdered sugar; this procedure left only the porous PDMS_PS sponge (see Figure S7).

After the preparations, all the systems were washed 3 times for 8 h in a mixture hexane:acetone 1:1 to completely remove any PDMS that was not polymerized. After the third wash, the FTIR spectra collected from the residues of the hexane/acetone mixtures indicated the absence of detectable PDMS residues.

In order to verify the absence of sugar residues the Fehling test was carried out onto 1 g of PDMS sponges fragments following the procedure reported in literature [49].

### 4.3. Physicochemical Characterization

FTIR spectra of the PDMS slabs and sponges were collected using a Shimadzu, Milan, Italy, IRAffinity-1S Fourier transform infrared spectrometer in transmittance mode (KBr pellets) for the liquid A and B components (Dow Sylgard™ 184) and using the MIRacle single reflection horizontal ATR accessory equipped with a diamond/ZnSe performance flat tip crystal plate. All spectra were then normalized for transmittance (%) intensity as a function of wavenumbers (cm^−1^). The resolution was 4 cm^−1^ and the number of scans was 64 for both the spectra collected in transmittance (KBr) and ATR mode. 

The SEM images were collected on a FEG-SEM ΣIGMA (Carl Zeiss, Jena, Germany) using an acceleration potential of 5 kV at a working distance of 5 mm. The metallization used gold vapor under vacuum; the magnifications were 50× and 100× for all the samples and there was no evidence of decomposition induced by the electron beam.

XMT analysis was carried out with a Skyscan 1172 high-resolution MicroCT system at CRIST Centre, University of Florence (Italy) on a sample of ~10 × 5 × 5 mm equipped with an X-ray tube having a focal spot size of 5 μm. The X-rays tube was equipped with a tungsten anode operating at 100 kV and 100 μA. 2D X-ray images (acquisition time is 3 s for each image) were captured over 180-degree on a rotating sample with a slice-to-slice rotation angle of 0.3 by placing the sample between the X-ray source and the CCD detector. The spatial resolution was ca. 5 μm. The 3D images were reconstructed from the tomographic projections using Micro Photonics Nrecon^®^ software version 1.6.10.4. Analysis of the obtained 3D images by means of the CTAn software v.1.18 allowed the pore size distribution of each sample subjected to XMT analysis to be obtained. Two different batches for each sample were considered and the error associated both to porosity and to the mean pores diameter was calculated by averaging these two parameters over the volume of the two samples.

The kinetics of solvent absorption (swelling and capillary action) of PDMS slab, PDMS_SC and PDMS_PS sponges were monitored by the weight increase of the slab and sponges as a function of time. PDMS slabs and PDMS_SC or PDMS_PS sponges were cut appropriately to obtain samples of 0.35 g (the exact weights *W_dry_*_(*time*=0)_ were determined on an analytical balance). The samples were kept immersed in 3 cm^3^ of each solvent in a capped vial and removed at appropriate time intervals. Then, the samples were softly and rapidly dabbed on a Whatman filter paper and immediately weighed (*W_wet_*_(*time*=*ti*)_). ATR-FTIR spectra were collected on the paper after its contact with the PDMS-based systems to determine whether part of the PDMS was removed during this operation. However, no evidence of the presence of PDMS residues was observed. The value of Weight Increase in percent (*WI*%)*_time_*_=*ti*_ was calculated according to the formula

$${\left(WI\%\right)}_{time=ti}=\frac{W_{wet\;(time=ti)}-W_{dry\;(time=0)}}{W_{dry\;(time=0)}}\times 100$$

This WI% was plotted as a function of time to obtain the kinetics curve of solvent absorption. The solvents tested were acetone (AC), ethanol (ET), isopropanol (PR), dimethyl-sulfoxide (DMSO), propylene carbonate (PC), water (W), diethyl carbonate (DC), cyclohexane (CH), ethyl acetate (EA). From these curves, the initial absorption rates were extracted as the slopes obtained by the linear fitting of the early-time absorption values, and the maximum amounts of the absorbed solvents were calculated from the asymptotic values of the curves reported.

The weight loss of the three classes of PDMS-based systems saturated with the solvents indicated above (the initial weight was *W_wet_*_(*time*=0)_) and placed in contact with sheets of Whatman^®^ paper, was monitored as a function of time. For these measurements, to minimize the evaporation of the solvents, the PDMS systems were covered with a polyester sheet (Melinex^®^). Evaporation of the solvent was monitored over time; even in this case, the PDMS systems were covered with a polyester sheet (Melinex^®^) and the evaporated solvent at time *t_i_* was measured too.

Then, the amount of solvent absorbed by the Whatman^®^ paper was calculated as follows:

$${∆W}_{TOT\;(time=ti)}={∆W}_{ABS\;(time=ti)}-{∆W}_{EVAP\;(time=ti)}$$

where:

$${∆W}_{TOT\;(time=ti)}=100-\left(\frac{W_{wet\;\left(time=0\right)}-W_{wet\;\left(time=ti\right)}^{\prime }}{W_{wet\;\left(time=0\right)}}\right)\times 100$$

$${∆W}_{ABS\;(time=ti)}=100-\left(\frac{W_{wet\;\left(time=0\right)}-W_{wet\;\left(time=ti\right)}^{\prime \prime }}{W_{wet\;\left(time=0\right)}}\right)\times 100$$

$${∆W}_{EVAP\;(time=ti)}=100-\left(\frac{W_{wet\;\left(time=0\right)}-W_{wet\;\left(time=ti\right)}^{\prime \prime \prime }}{W_{wet\;\left(time=0\right)}}\right)\times 100$$

Here $W_{wet\;\left(time=ti\right)}^{\prime }$ is the total weight loss, $W_{wet\;\left(time=ti\right)}^{\prime \prime }$ is the weight loss due to the solvent absorption by the Whatman^®^ paper and $W_{wet\;\left(time=ti\right)}^{\prime \prime \prime }$ is the weight loss due to solvent evaporation.

The rheology measurements were carried out using a TA Discovery HR-3 hybrid rheometer according to the following procedure. Frequency sweep measurements were performed in the linear viscoelastic range, to monitor the behavior of the two parameters G′ (elastic modulus) and G″ (viscous modulus) as a function of the oscillation frequency at constant oscillation amplitude. The check of this range was determined through amplitude sweep measurements of G′ and G″ at constant frequency sweep (1 Hz) as a function of the oscillation amplitude (Figure S8). The normal force was set equal to 0.5 N for all measurements. The evaporation of the solvent was minimized by using a solvent trap system provided by TA Instruments (New Castle, DE, USA). All the measurements were repeated at least three times in order to verify the reproducibility. 

### 4.4. Application Tests

In order to determine the efficacy of this methodology, two different cleaning tests were carried out using a PDMS_PS sponge soaked with acetone onto the surface of a fresco mock-up coated with a 20 years old layer of poly(EMA/MA) 70:30 and a canvas painting mock-up coated with a ketone resin. Two different cleaning procedures were followed: for the fresco, three applications on three different areas were carried out on the fresco surface with the following contact times: 1 min, 5 min and 12 min (a small sample of the cleaned area was collected of the uncleaned area and after each application), while a single application was carried out on the canvas sample with a contact time of 12 min. 

FTIR spectra of samples from the fresco mock-up were collected in transmittance mode using the following procedure: four small (few mg) fragments of fresco, collected before the cleaning and after each application of the PDMS_PS sponge soaked with acetone, were immersed in chloroform for 24 h in order to selectively extract the poly(EMA/MA) 70:30 film residues. Then, 25 drops of the resulting solutions were placed into an agate mortar and the solvent allowed to evaporate. The spectrum of the obtained solid residue was recorded using a pellet made from 200 mg of KBr containing 0.0125 wt% of the residue and Prussian blue as internal standard. The Prussian blue was added in order to normalize the intensity of the peak at 1732 cm^−1^ (that is a marker of the poly(EMA/MA) 70:30) with the Prussian blue signal at 2092 cm^−1^. This allowed a semiquantitative comparison among the different samples [57]. Spectra are the average of 64 scans recorded in the absorbance mode using a BioRad (Milan, Italy) model FTS-40 spectrometer with a resolution of 4 cm^−1^.

The FTIR spectra of the area of interest on canvas in the cleaning tests with the PDMS_PS sponge soaked with acetone were collected in ATR mode through the procedure described above. 

## Figures and Tables

**Table 1:** Porosities and mean pore diameters (mm) of the pores of PDMS_SC and PDMS_PS sponges as calculated from the % of polymers.

Sample	Polymer (%)	Porosity (%)	Mean Pore Diameter (µm)
PDMS_SC	22.6 ±0.7	77.4 ± 5.2	303 ± 15
PDMS_PS	90.2 ±1.5	9.8 ± 0.38	76 ± 5

## Data Availability

The data presented in this study are openly available in article.

## Supplementary Materials

The following supporting information can be downloaded at: https://www.mdpi.com/article/10.3390/gels9120985/s1. Figure S1: Frequency sweep diagram of the PDMS sponges; Figure S2: Scanning Electron Microscopy (SEM) images collected before and after the application of PDMS_PS sponge loaded with ca. 10 wt% of EA onto the surface of a fresco mock-up; Figure S3: Scanning Electron Microscopy (SEM) images collected in a region of the canvas painting mock-up where the coating was absent and in a region affected by the surface coating before and after the application of PDMS_PS sponge loaded with ca. 10 wt% of EA; Figure S4: Photographs of the obtained PDMS slab; Figure S5: Trend of the elastic modulus G′ for a mixture composed by the base and by the curing agent of the Sylgard 184^®^ kit as a function of time; Figure S6: Photograph of the obtained PDMS_SC sponge; Figure S7: Photograph of the obtained PDMS_PS sponge; Figure S8: Amplitude sweep diagram of the PDMS based systems.
