# Supplementary material for: Non-Aqueous Poly(dimethylsiloxane) Organogel Sponges for Controlled Solvent Release: Synthesis, Characterization, and Application in the Cleaning of Artworks
Source: Gels. 2023 Dec 15;9(12):985. doi: 10.3390/gels9120985 (PMC10742450; doi:10.3390/gels9120985)
Supplement: Supplementary file 1 [file gels-09-00985-s001.zip › gels-2739361-supplementary.pdf]

Supplementary Materials

Non-Aqueous Poly(dimethylsiloxane) Organogel Sponges for Controlled Solvent Release: Synthesis, Characterization, and Application in the Cleaning of Artworks

Francesca Porpora [1], Luigi Dei [1], Teresa T. Duncan [2], Fedora Olivadese [1], Shae London [3], Barbara H. Berrie [4], Richard G. Weiss [3] and Emiliano Carretti [1,5,*]

[1] Department of Chemistry "Ugo Schiff" & CSGI Consortium, University of Florence, Via della Lastruccia, 3-13, 50019 Sesto Fiorentino, Italy; francesca.porpora@unifi.it (F.P.); luigi.dei@unifi.it (L.D.); fedora.olivadese@stud.unifi.it (F.O.)

[2] Scientific Analysis of Fine Art, LLC, Berwyn, PA 19312, USA; ttd5@georgetown.edu

[3] Department of Chemistry and Institute for Soft Matter Synthesis and Metrology, Georgetown University, 37th and O Streets NW, Washington, DC 20057, USA; ssl63@georgetown.edu (S.L.); weissr@georgetown.edu (R.G.W.)

[4] Department of Scientific Research, National Gallery of Art, 2000 South Club Drive, Landover, MD 20785, USA; b-berrie@nga.gov

[5] National Research Council—National Institute of Optics (CNR-INO), Largo E. Fermi 6, 50125 Florence, Italy

* Correspondence: emiliano.carretti@unifi.it; Tel.: +39-0554-573-046

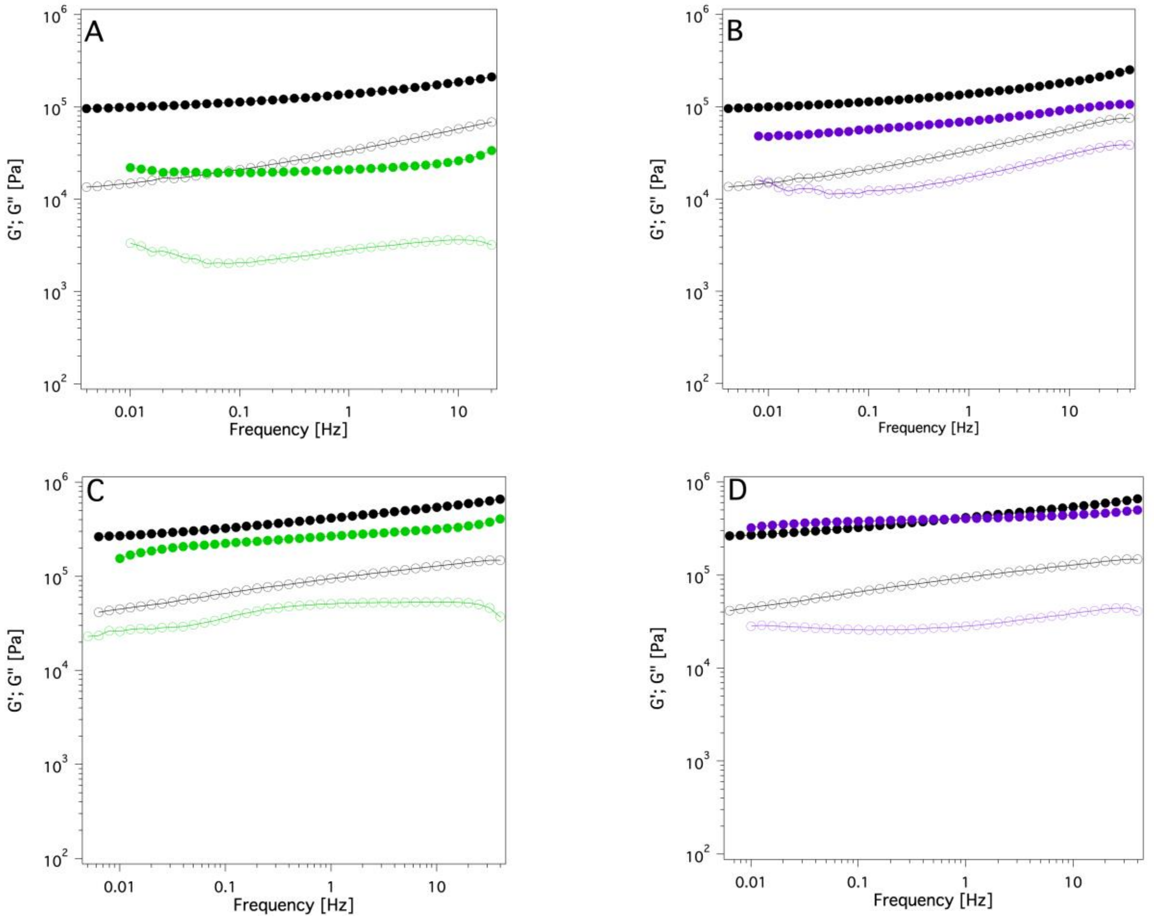

Figure S1. Frequency sweep diagram of the: (**A**) PDMS_SC sponge alone (black), with absorbed Ethyl acetate (green); (**B**) PDMS_SC sponge alone (black), with absorbed Ethanol (purple); (**C**) PDMS_PS sponge alone (black), with absorbed Ethyl

acetate (red); D PDMS_PS sponge alone (black), with absorbed Ethanol (purple). Absorption time 24 hours. Filled circles indicate G′; open circles indicate G″.

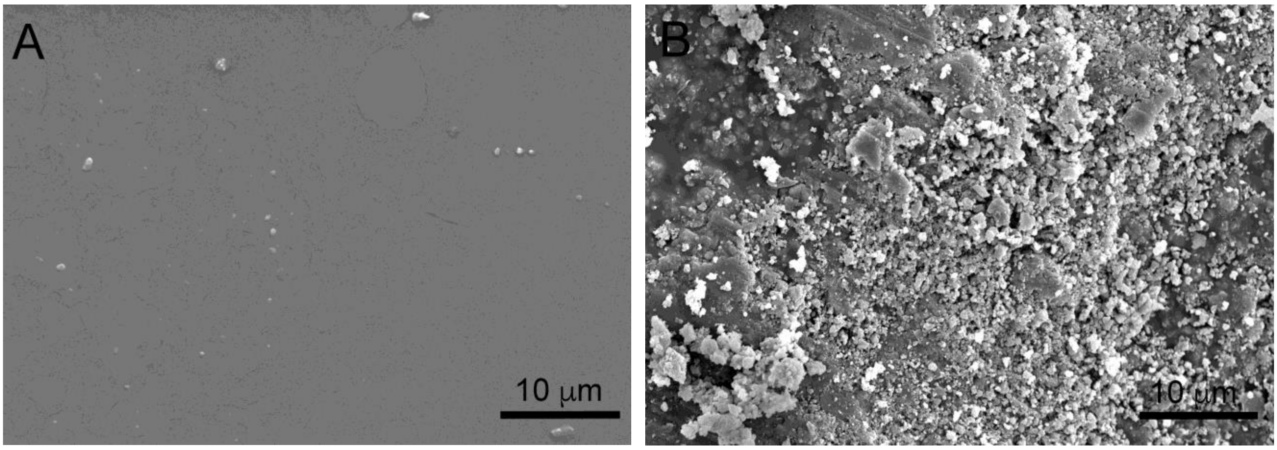

Figure S2. Scanning Electron Microscopy (SEM) images collected before (**A**) and after (**B**) the application of PDMS_SP sponge loaded with 10wt% ca of EA onto the surface of a fresco mockup.

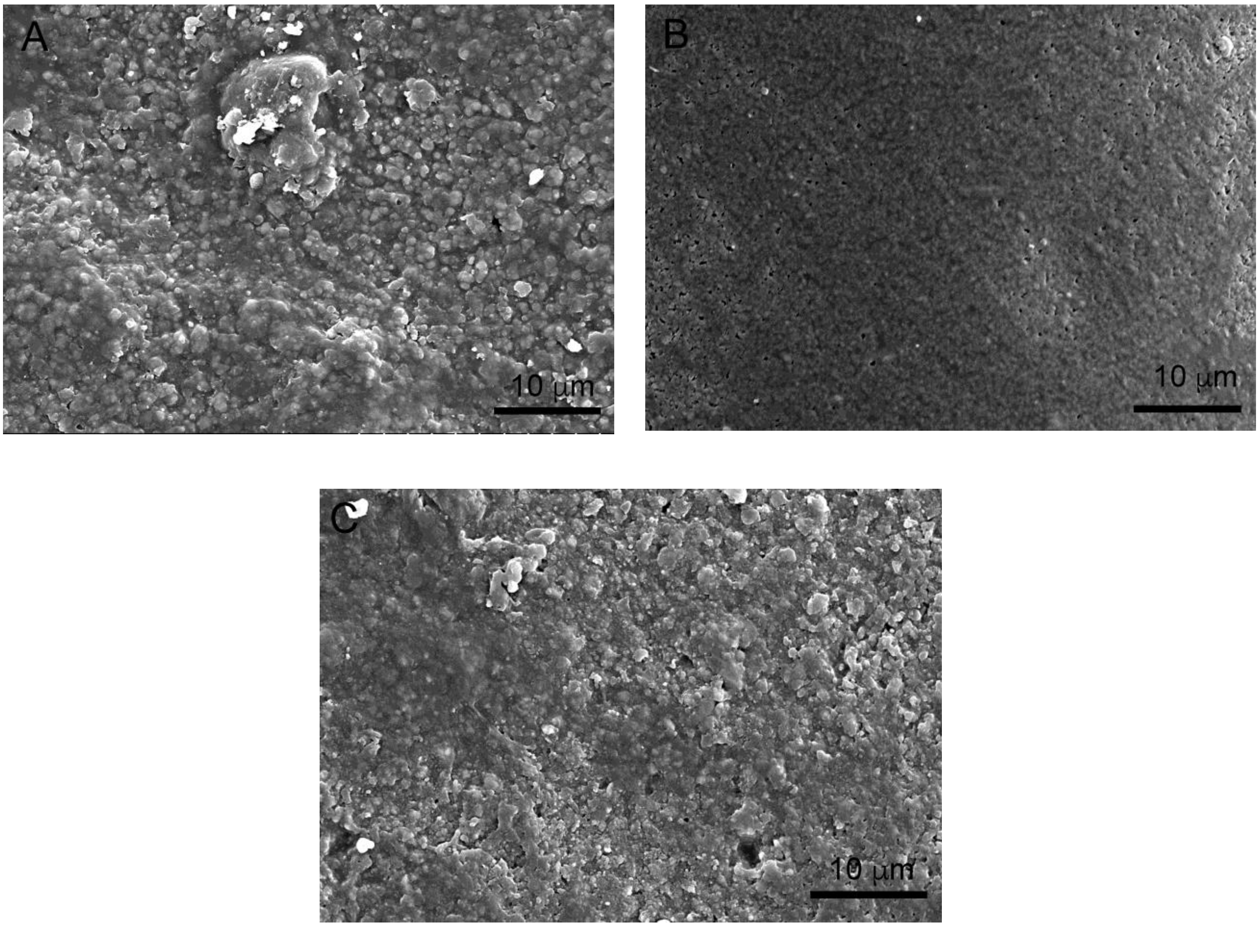

Figure S3. Scanning Electron Microscopy (SEM) images collected in a region of the canvas painting mockup where the coating was absent (**A**) and in a region affected by the surface coating before (**B**) and after (**C**) the application of PDMS_SP sponge loaded with 10wt% ca of EA.

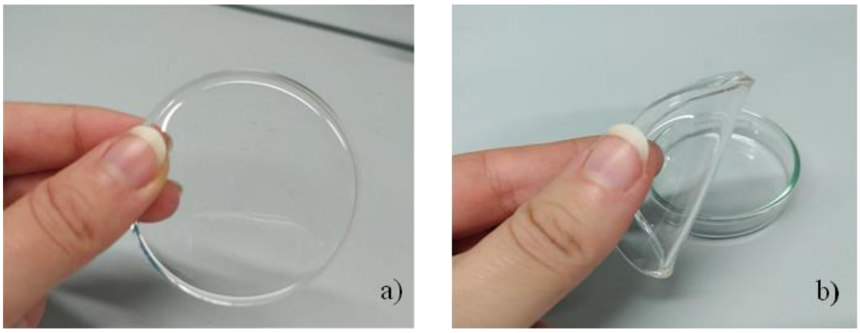

Figure S4. Photographs of the obtained PDMS slab showing both transparency (**a**) and elasticity (**b**).

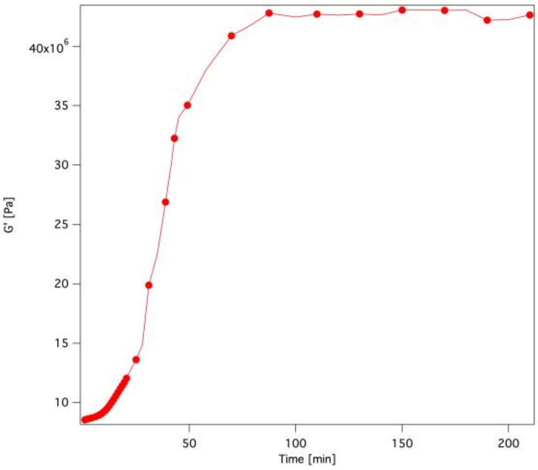

Figure S5. Trend of the elastic modulus G′ for a mixture composed by the base and by the curing agent of the Sylgard 184® kit (ratio component A/component B = 20:1) as a function of time at constant frequency (1Hz), amplitude strain (1%) and temperature (60 °C).

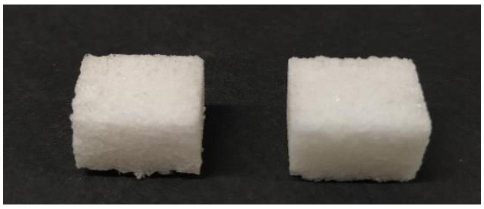

Figure S6. Photograph of the obtained PDMS_SC sponge (left) compared with the pure sugar cube (right).

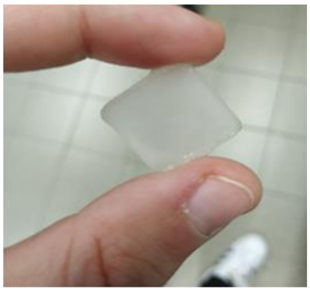

Figure S7. Photograph of the obtained PDMS_PS sponge.

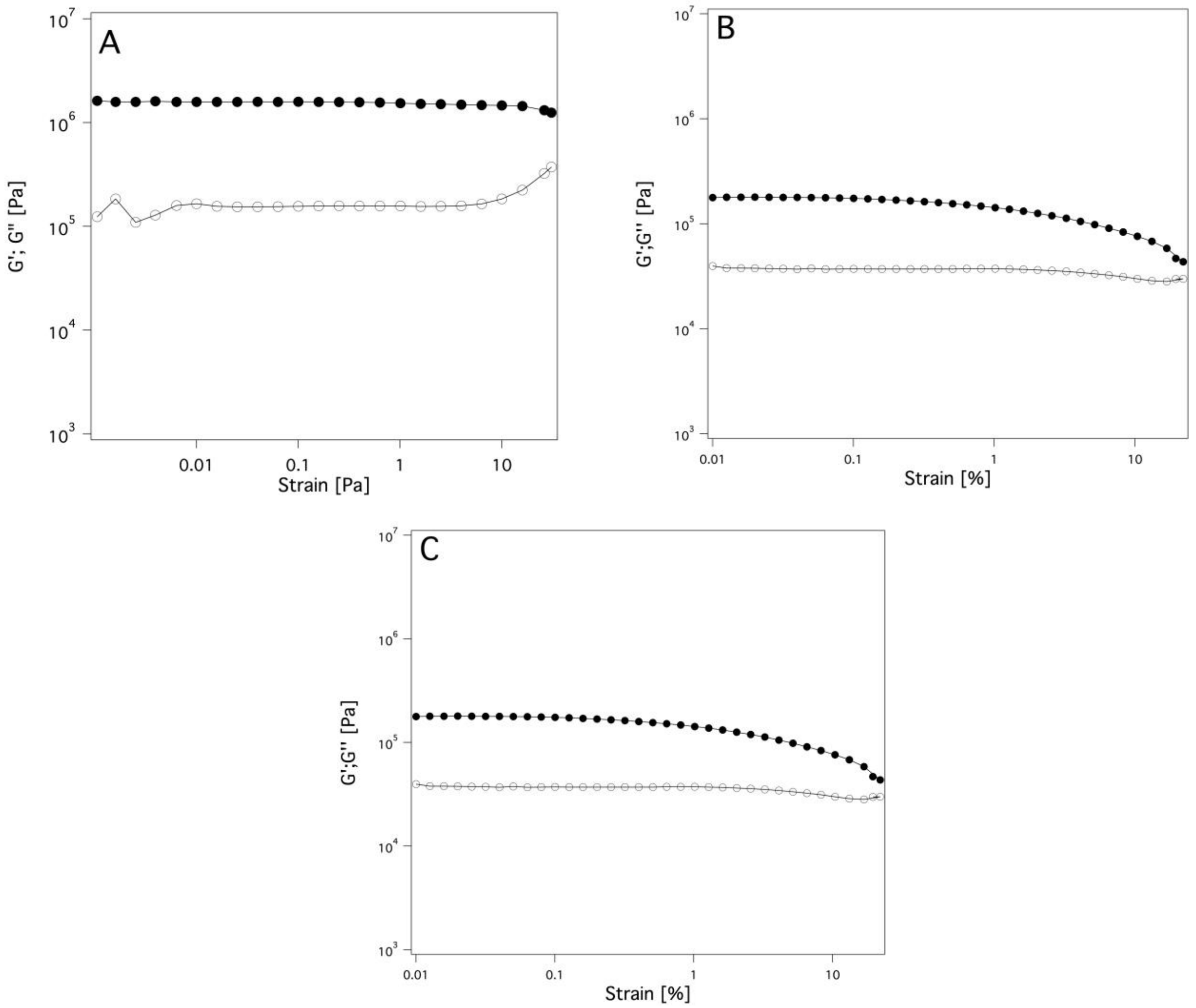

Figure S8. Amplitude sweep diagram of the: (**A**) PDMS slab 10:1; (**B**) PDMS_SC sponge (ratio component A/component B = 10:1); (**C**) PDMS_SP sponge (ratio component A/component B = 10:1). Filled circles indicate G′; open circles indicate G″.
